# Medicinal Plant Analysis: A Historical and Regional Discussion of Emergent Complex Techniques

**DOI:** 10.3389/fphar.2019.01480

**Published:** 2020-01-09

**Authors:** Martin Fitzgerald, Michael Heinrich, Anthony Booker

**Affiliations:** ^1^ Herbal and East Asian Medicine, School of Life Sciences, College of Liberal Arts and Sciences, University of Westminster, London, United Kingdom; ^2^ Pharmacognosy and Phytotherapy, UCL School of Pharmacy, London, United Kingdom

**Keywords:** herbal medicine, medicinal plant, analysis, quality, pharmacopoeia, complexity, advances

## Abstract

The analysis of medicinal plants has had a long history, and especially with regard to assessing a plant’s quality. The first techniques were organoleptic using the physical senses of taste, smell, and appearance. Then gradually these led on to more advanced instrumental techniques. Though different countries have their own traditional medicines China currently leads the way in terms of the number of publications focused on medicinal plant analysis and number of inclusions in their Pharmacopoeia. The monographs contained within these publications give directions on the type of analysis that should be performed, and for manufacturers, this typically means that they need access to more and more advanced instrumentation. We have seen developments in many areas of analytical analysis and particularly the development of chromatographic and spectroscopic methods and the hyphenation of these techniques. The ability to process data using multivariate analysis software has opened the door to metabolomics giving us greater capacity to understand the many variations of chemical compounds occurring within medicinal plants, allowing us to have greater certainty of not only the quality of the plants and medicines but also of their suitability for clinical research. Refinements in technology have resulted in the ability to analyze and categorize plants effectively and be able to detect contaminants and adulterants occurring at very low levels. However, advances in technology cannot provide us with all the answers we need in order to deliver high-quality herbal medicines and the more traditional techniques of assessing quality remain as important today.

## Introduction 

Medicinal plants have been a resource for healing in local communities around the world for thousands of years. Still it remains of contemporary importance as a primary healthcare mode for approximately 85% of the world’s population (Pešić, 2015), and as a resource for drug discovery, with 80% of all synthetic drugs deriving from them (Bauer and Brönstrup, 2014). Concurrently, the last few hundred years has seen a prolific rise in the introduction, development, and advancement of herbal substances analysis. Humans have been identifying and selecting medicinal plants and foods based on organoleptic assessment of suitability and quality for thousands of years, but it is only in the span of the last seven decades since the invention of basic analytical techniques, e.g., paper chromatography, that has seen rapid development from sight, touch, and smell to using sophisticated instrumentation. Though this mechanization of the senses has appeared relatively recently, historically conceptual expansion has been building throughout the scientific revolution, outwards toward the universe and inwards to a scale below recognition capable with a human eye, leading to development of some of the earliest analytical tools assisting the senses, the telescope and microscope. From the initial discovery of new microscopic worlds, through structural, chemical, and atomic levels, the sensitivity and range of human perception has been extended and enhanced.

Rapid progress is especially evident considering that the concept of a laboratory was only formally formed in Europe during the early 1600s. First as an extension of philosophers’, doctors’, and scientists’ workrooms, it becomes a space to study nature and gather empirical evidence (Wilson, 1997), where studies could be conducted at the analyst’s convenience rather than at specific times when daylight or weather permitted. This was a small but important step towards more formalized analytical investigations.

In modern analysis, single techniques such as paper chromatography and much earlier colorimetry appeared. It was followed by a greater range and wider application of these techniques until early hyphenations such as LC-UV emerged, culminating more recently in multiple combinations of multi-hyphenated instrumentation, availing of the analytical advantages inherent in each individual technique. The emergence of hyphenated analytical techniques in many aspects is analogous to the organoleptic synthesis that occurs when selecting a medicinal plant; viewing, smelling and tasting it to use combinations of different senses, increasing the points of reference/statistical degrees of freedom to improve the probability of correctly identifying and assessing its quality. The emergence and application of these hyphenated techniques only became possible and useful as computer systems and data management tools developed, enabling rapid and selective synthesis of information from the large amount of instrumental and analytical data signals generated.

Probably the single greatest influence in recent times in the advancement of the analysis of herbal materials (and arguably analysis generally) is, though, how large amounts of data can be collected, assimilated, and used more meaningfully in human readable forms. Similar to the historical advancements in combinatorial hyphenated instrumentation, now combinatorial data processing techniques like fingerprinting, metabolomic profiling, and pattern recognition algorithms have emerged, further increasing analytical capabilities, while reducing operator time and expertise required. This trend has further accelerated the pace and rate of advancement of analytical techniques and has led to an increase in the pace and capability of the associated research. In this paper, we analyze publication trends and pharmacopoeial developments in order to better understand the role and progression of analytical techniques. Since their initial discovery and development, with a particular focus on China, an Asian country with both deep cultural and long-term historical roots in plant medicine, to more modern day developments and applications.

## Publication Trends

**Figure 1:**
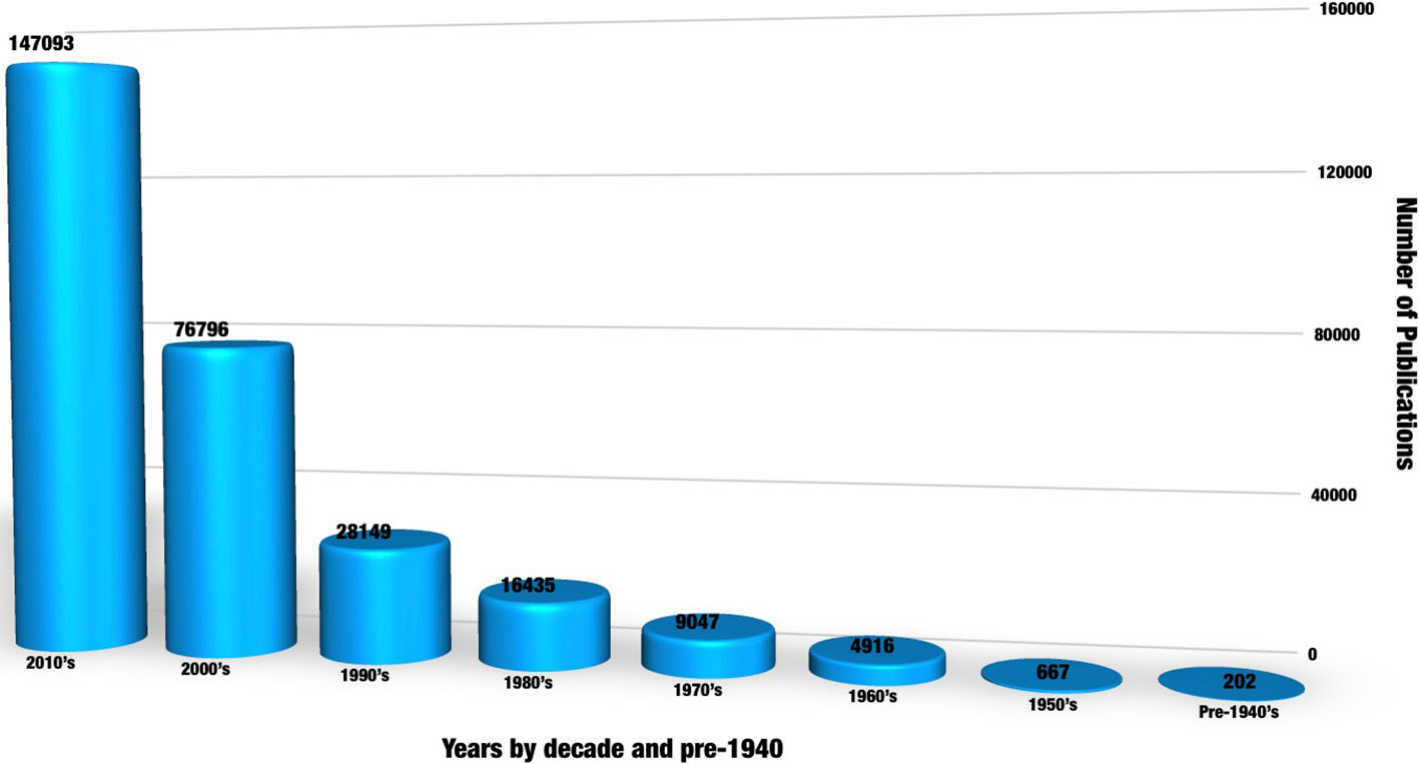
The herbal substance analysis publications trend since search records began in 1800. A keyword search was conducted using the combination “medicinal plant” OR “herbal medicine” AND “analysis” chosen for the maximum retuned records after exploring a list of similar topic and combination of keywords such as “photochemical analysis,” “traditional medicine,” and “herbal.” The Web of Science or collection, KCI- Korean Journal database, MEDLINE^®^, Russian Science Citation index, and SciELO Citation index databases were included in the search.

Increasing interest in medicinal plant research and analysis is reflected in the number of recent publications, with more than a three-fold increase from 4,686 publications during the year 2008 to 14,884 in 2018. Output published during the 8 years of the present decade alone outnumbered all those combined before 2000, since the included database records began in 1800 (**Figure 1**).

**Figure 2:**
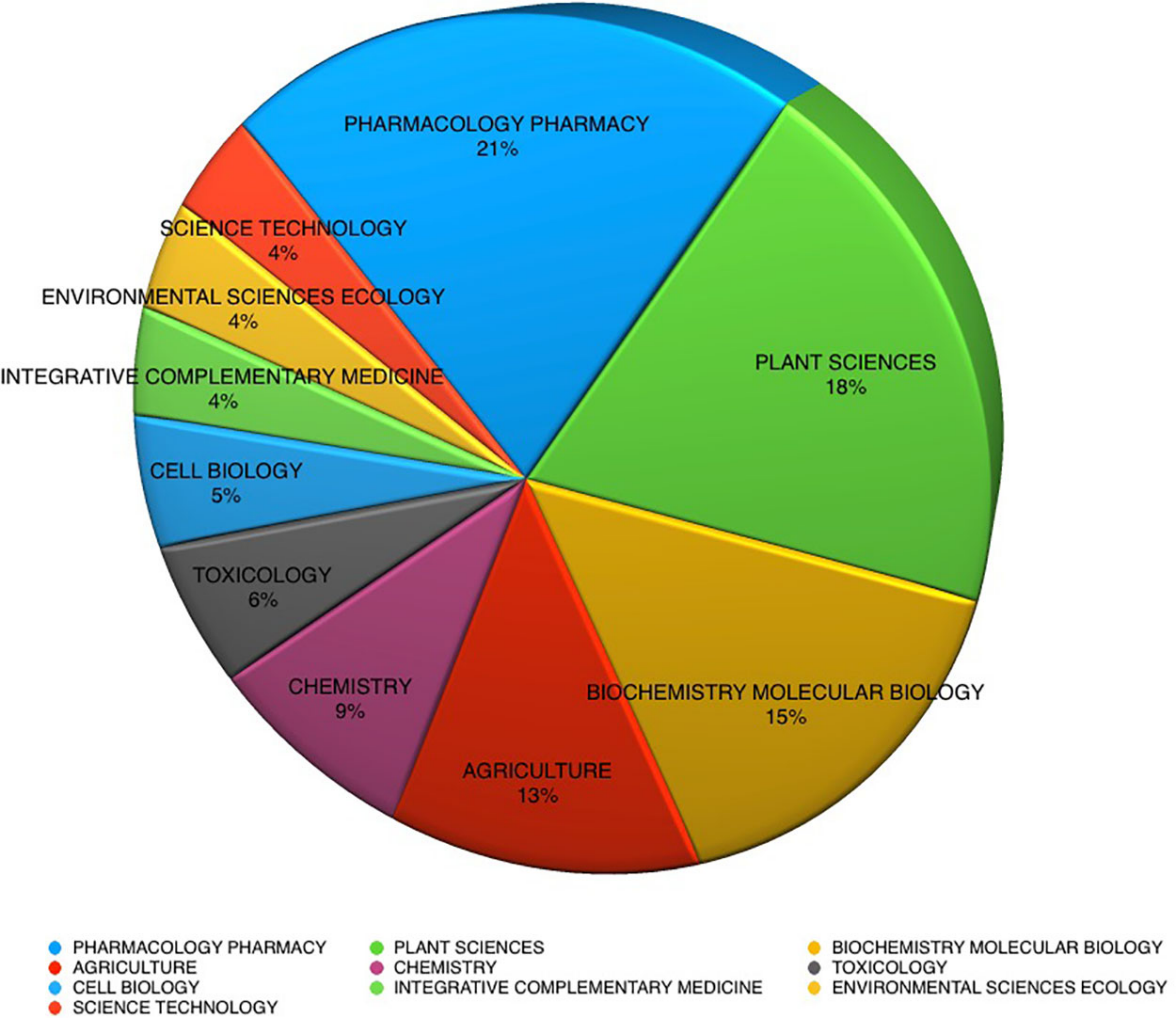
Herbal substance analysis publications by discipline, 2008–2018 (169,917 records).

The largest proportion of publications cited in current databases over the last 10 years for medicinal plant analysis reports are in the disciplines of pharmacology and pharmacy (**Figure 2**). With plant sciences, biochemical molecular biology and agriculture research following closely behind, together comprising almost 70% of the total publications.

## Regional Trends—Last 10 Years

**Figure 3:**
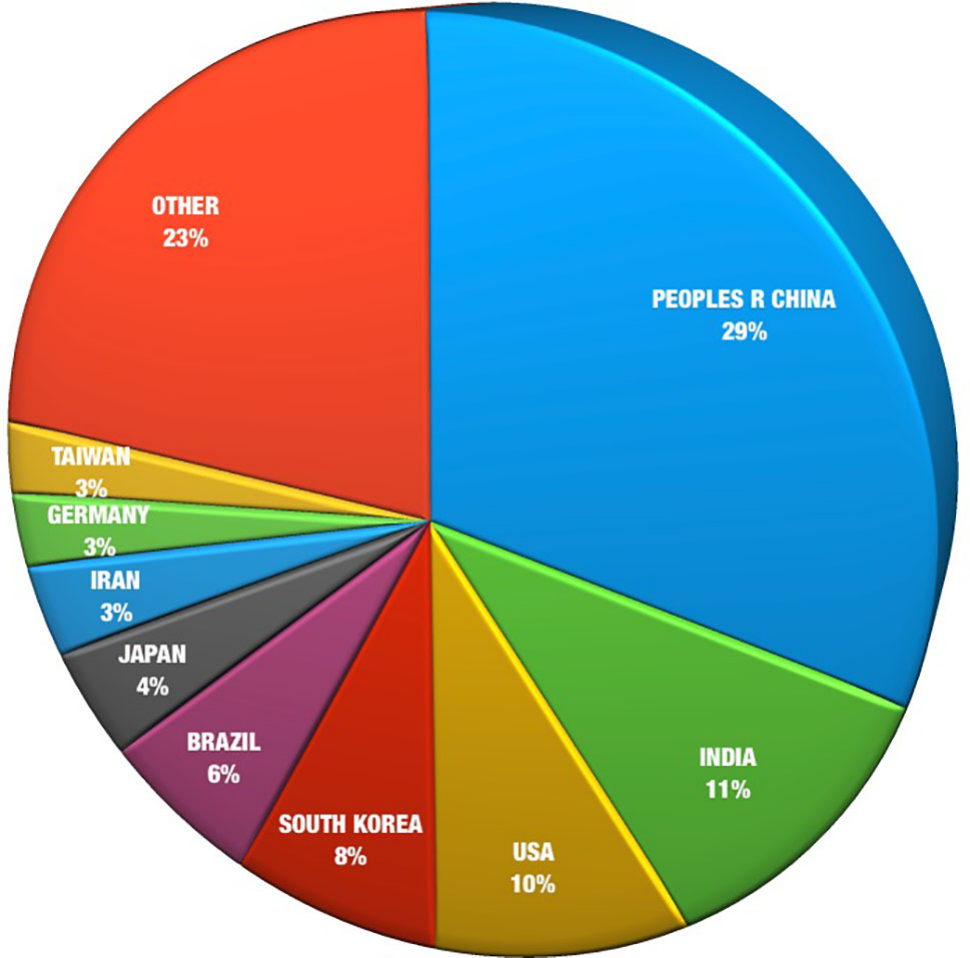
Herbal analysis publications by region, 2008–2018.

The majority (about 58%) of medicinal plant analysis publications in the last 10 years have collectively emerged from mainland China, India, USA, and South Korea (**Figure 3**). This may be an expression of the strong medicinal plant traditions in Asia in addition to the USA’s dominant presence as an international user of herbal products (Hu et al., 2013). The major East Asian regions, in particular, China, Japan, South Korea, together with Taiwan, contribute more than half of the total citations (55%). This may be indicative of the rapid economic progress and technological capability of these countries. China is the major contributor, with a 15% increase in its dominance of research outputs in the last 10 years. This influence has also been seen in the effect of China’s growing involvement in aiding the development of pharmacopoeias around the world and as a leader in the analysis of Chinese medicinal plants (**Figure 3**).

## Regulation and a Changing Analytical Landscape

From a regulatory perspective, the pharmacopoeial requirements are the central reference point for the analysis of medicinal plants. Though internationally many pharmacopoeias exist, the most comprehensive of these relating to herbal medicinal materials is the Chinese Pharmacopoeia (ChP). The current ChP introduced in 2015 is the 10th iteration presented in three volumes and includes 5,608 drugs, a 10-fold increase from its first edition in 1953. More than half of the current monographs (Hamid-Reza et al., 2013, 598) relate to CHM specifically including raw plants, slices, herbal mixtures, and oils. A noticeable inclusion in the current version compared with the previous version is the addition of 400 herbal mixtures (Qian et al., 2010).

## Pharmacopoeia Monographs—Their Influences and Challenges

Though more recently the ChP is playing an increasing role in influencing medicinal plant analysis, the development of the ChP has been heavily influenced by Western pharmacopoeias. Historically the identification, preparation, and analysis of medicinal plants were based on classic texts such as the Shengnong Bencao Jing (Shengnong Materia Medica, 25–220 CE), where the category and quality of 365 plants and 113 prescriptions were assessed by taste. Organoleptic sensing of bitterness, sweetness, saltiness, and even neutral tastes were thought to indicate the function and application of the medicine. Arguably, the most influential Chinese pharmacy monograph is the Bencao Gangmu (Compendium of Materia Medica, 1368–1644 CE) containing 1,892 plant descriptions and 11,096 prescriptions sorted in 16 divisions and 60 orders, emphasizing appearance, taste, and odor as a key to authentication and quality.

However, the main precursor to the modern format of the current Chinese Pharmacopoeia was printed in the 1930s with 670 drugs. Even at this early stage, the then dominant Western powers such as Britain, Germany, America, and Japan found challenges in understanding and forming consensus for recognizing, categorizing, and assuring the quality of Chinese medical materials. At this time a difficulty emerged in securing materials for the more Western styled “scientifically run” hospitals. Initially it was though that as Japan had adopted a translation of the German pharmacopoeia in 1886, the Chinese could follow suit using the British Pharmacopoeia, which in 1927 had been translated into Chinese as a joint effort by the London and British Chambers of Commerce. However, some differences in opinion between the four occupiers had to be first resolved.

Many of the technological demands necessary to produce and maintain the pharmacopoeial standards required by the Americans was beyond the ability and technological capability of the Chinese at that time. America had recently just printed a Chinese translation of its United States Pharmacopeia (10^th^ edition) published in 1926. The strict American standards for aconite, digitalis, adrenalin, and insulin were purported to be managed by new or foreign trained pharmacists (Read, 1930). Preparations such as liniments found in the British and U.S. Pharmacopoeias were included in the Chinese version. Syrups such as those of codeine and glucose and tinctures of cannabis were from the British influence. Foreign residents in China found it difficult to ingest local food and stated an “extensive need for bowel remedies.” Therefore, drugs of the time, albuminis, aspidium, and emetin, were included. Vaccines for diphtheria, tetanus, and smallpox were maintained through the instruction of the USP.

German chemists had already gained a reputation for the isolation of chemical compounds, many of which were used medicinally and were already included in the Japanese Pharmacopoeia such as oxalic acid, pyrogallic acid, and bromine. Therefore, the existing German-Japanese analytical methods were generally utilized for these areas, which comprised about 25% of the new Chinese Pharmacopoeia. Whereas more British and American derived analytical methods and preparations were included for vegetable- and animal-based materials.

Agreement over the correct translation and naming of chemical compounds also proved problematic, e.g. when attempting to resolve disagreement between German-Latin and Anglo-American descriptions such as “natrium chloratum” and “sodii chloridum.” The shared Latin common language elements aided European and American common understanding; however, translation into Chinese was troublesome. A potentially easier route would have been to adopt the Japanese Pharmacopoeia names and descriptions, often possessing the same Asian (Hanzi) character as that in China, however, this was resisted due to the strong nationalistic sentiment at the time in mainland China (Read, 1930).

Though the Japanese favored direct foreign phonetic transliterated terms for drugs, about 60 original Chinese *materia medica* entries had persisted in the Japanese Pharmacopoeia including entries for camphor, ginger, aloes, cardamom, and star anise.

Difficulty in plant identification and common naming was not confined to Asia. During the early 1900s period of European and American political expansion, attempts were being made in Europe to catalogue multilingual terms for similar plants such as the publication of “the illustrated polyglot dictionary of plants names” in Latin, Arabic, Armenian, English, French, German, Italian, and Turkish languages (Bedevian, 1936), cataloguing 3,657 plants in eight languages.

## Chronology of Pharmacopoeial Developments in China

### 1900–1949

Medicinal plant publications during the early 1900s, before the formation of the People’s Republic of China in 1949, were greatly influenced by the previous “age of exploration.” Many scientific societies were set up by explorers, their peers, and investors as forums to communicate knowledge and acknowledge ownership of findings and discoveries (Fyfe and Moxham, 2016). The rise in fashion of the “gentleman scholar” engaging in academic pursuits supported the occupation of writing. During this time, many publications focused on the identification and classification of ethnic/indigenous medical plants, such as Aztec medicinal plants still in use in modern Mexico (Braubach, 1925; Heinrich et al., 2014), Algonquians from nowadays, Canada, (Speck, 1917), Micronesians (St John, 1948), Babylonians and Assyrians, (Jastrow, 1914), Native American Indian tribes (Castetter et al., 1935), Persia, (Garrison, 1933) and India, (Chopra, 1933). Publications in English describing the history and use of Chinese medicine in the context of Western orthodox also appeared (Chan, 1939).

### Post-1949

Periods of advancements in TCM research after 1949 to the present day have been described as occurring in three defined phases lasting about 20 years each. The first was 1950–1970, springing from the rapid development of TCM in universities, research, and hospitals in China during this time. The second phase took place during 1980–2000s, where we see the construction of legal, economic, and scientific networks. The third phase, from 2000 to date, is defined by a focus on elucidating the scientific basis and scientific clinical practice of TCM using cross-disciplinary and global collaborations (Xu et al., 2013).

### 1950–1969

#### Political Context

This period immediately followed the formation of the People’s Republic of China and saw a rise in nationalism and political introspection. International relationships cooled and a closer connection with the Soviet Union was officially forged with the Sino-Soviet Treaty of Friendship, Alliance, and Mutual Assistance in 1950.

#### Regulatory and Pharmacopoeial Developments

This period saw the launch of the first edition of the People’s Republic of China Pharmacopoeia (ChP) in Chinese launched in 1953. It contains 531 monographs and mainly retains the information of the previous precursor published in the 1930s, compiled from foreign influences. It guided both identification and quantification of synthetic drugs and medicines together in one issue. Some crude herbal materials were listed, but not in analytical detail. Internationally post-World War II, good-will fostered a sense of cooperation and collaboration. This was also reflected by the World Health Organization’s release of the international pharmacopoeia (Ph. Int) issued by the World Health Organization in 1951, produced in two volumes. It contained 344 monographs and 84 tests, with an aim to provide a harmonized international reference for pharmacopoeial methods. The first European Pharmacopoeia Ph. Eur. was produced in 1967, with a more European focus, but combining many common elements of the long-existing British Pharmacopoeia and the United States Pharmacopeia.

#### Medicinal Plant Research and Analytical Development

Research publication output during the 1950s was varied but the most cited publication trends concerned identification of plant species using electron microscopy (Watson, 1958), the use of plant tissue staining methods (Bergeron and Singer, 1958; Fernstrom, 1958), and use of plant extracts for colorimetric analysis (Holt and Withers, 1958; Lillie, 1958). Though originating in the 19^th^ century, the analytical tradition of extraction, purification, and separation of chemical plant components, e.g., the alkaloids, became increasingly sophisticated during this period (Svoboda et al., 1959). Toxicity studies during this time were still basic, exposing mainly mice to plant extracts and using mortality rate counting and organ biopsy and cell station techniques, e.g., quercetin, podophyllotoxin, and podophyllin extract toxicity studies (Leiter et al., 1950) and induced liver lesions with Pyrrolizidine alkaloid extracts (Schoental, 1959).

Chemical screening of plants for their medicinal effects in various chemical and clinical trials is featured (Farnsworth, 1966) as did their use in derivatized forms for the treatment of nerve inflammation (Jancso et al., 1967) and in human metabolism studies (Pletscher, 1968). Studies into the use of medicinal plants for their potential use in cancer treatments were encouraged by the first isolation of paclitaxel from the pacific yew, *Taxus brevifolia* Nutt.

Older basic chromatographic techniques that had been already in use remained commonly used analytical techniques, e.g., paper chromatography applied to the analysis of common broom [*Cytisus scoparius* (L.) Link.] (Jaminet, 1959) and in medicinal plant quality control (Paris and Viejo, 1955). Separation of alkaloids e.g. in *Duboisia myoporoides* R. Br. (Hills and Rodwell, 1951) remained a common interest and the analysis of other important metabolites including scilliroside in red squill, *Drimia maritima.* (L.) Stearn (Dybing et al., 1954). An investigation of *Cannabis sativa* L. for its antibacterial activity was also conducted during this timeframe (Krejci, 1958).

Much of the medicinal plant research of this period concerned the extraction and isolation of single compounds from plants. Basic colorimetric tests, UV-visible and infrared spectroscopy, and paper chromatography had previously supported this type of analysis. Spectroscopic techniques such as UV-Vis spectrometry with chart recorders had been in use since the 1920s (Hardy, 1938). These were being increasingly used for quantitative applications, such as in the analysis of glucoside in walnuts and monitoring the chemical composition of plants in relation to seasonal variations (Daglish, 1950).

However, the 1950–1970s was a golden period for the development of analytical technology. A time when the techniques of mass spectrometry (MS), nuclear magnetic resonance (NMR) spectroscopy, and gas chromatography (GC) techniques had come of age. Mass spectrometry, which had been invented in the late 1800s and used in a more analytical form during the 1910s, had now come into a relatively more advanced era. It was during the period 1950–1970 that the ion trap technique was developed, for which Dehmelt and Paul later received a Noble prize. The Purcell and Bloch groups at Harvard and Stanford University, respectively, developed NMR techniques and in 1952 also received a Nobel Prize (in Physics). In 1952, Archer John Porter Martin and Richard Synge also shared a Nobel Prize (in chemistry) for inventing partition chromatography, the basis of modern GC. Gas–liquid separations solved the problem of separating sugar-based molecules, which tended to bond with traditional stationery phases such as silica and volatile compounds, such as volatile oils, which are lost through evaporation during collection, preparation, and analysis. GC was applied for the first time to resolve 17 difficult to separate plant glycosides from a broad range of chemical classes, including phenolic, coumarin, isocoumarin, isoflavone, anthraquinone, cyanogenic, isothiocyanate, and monoterpene (Furuya, 1965), 15 kinds of valerian sesquiterpenoids in valerianaceous plant oils (Furuya and Kojima, 1967), and the extraction and analysis of rose oil (Minkov and Trandafilov, 1969).

Publications included well-applied examples where visible, ultra-violet (UV), and infrared (IR) spectral data were combined to elucidate structural characteristics of plants while undergoing chemical degradation, e.g., the stereochemical discrimination of lignin components paulownin and isopaulownin from *Paulownia tomentosa* Steud. (Takahashi and Nakagawa, 1966), the alkaloids of the Orchidaceae (Lüning et al., 1967), and terpenoids of *Zanthoxylum rhetsa* DC (Mathur et al., 1967).

MS was also used side-by-side with NMR, resulting in the structural elucidation of key metabolites, e.g., the characterization of the opium papaverrubine alkaloids and their *N*‐methyl derivatives in the genus *Papaver* (Brochmann-Hanssen et al., 1968), the analysis of three new coumestan derivatives from the root of licorice, *Glycyrrhiza* spp., (Shibata and Saitoh, 1968), and the isolation and purification of polyprenols from the leaves of *Aesculus hippocastanum* L. (horse chestnut) (Wellburn et al., 1967).

Up to this time, China had played a very marginal role in international research and development activities, a situation that was to change significantly in the following period.

### 1970–1989

#### Political Context

1971 saw China’s introspection from the Mao era revert to more external international engagement with the “People’s Republic of China” (PRC) elected as a permanent member of the United Nations’ General Assembly. This followed the American government’s extension of political relations with PRC after the Richard Nixon presidential visit that catalyzed an “Opening up to the West” phase in Chinese history. This opening began in 1978, orchestrated by the interim leader Deng Xiaoping, who initiated support for wide sweeping economic reforms. On a local level this manifested as individuals within China being allowed to make personal economic decisions, with the tightly governed communes being dissolved. Rural markets were replaced by open markets, resulting in a dramatic increase in international trade, supporting Xiaoping’s wish to fund economic growth from foreign investment. In the context of medicine, China’s ambition to look outward was highlighted over a decade earlier by a University College London anatomy Professor, Derrick James, when a British delegation visited China in 1954 and in his subsequent Lancet article outlined China’s intention to introduce a more scientific, modernized TCM (James, 1955).

As international trade from China expanded, so did the trade in medicinal plants from Asia and with it, increased access for Chinese scientists to modern analytical instrumentation. Internally by the mid-1980s, 25 Chinese medicine colleges were formed in a reportedly scientific and modern style with an almost 30-fold increase of TCM hospital beds to 2.5 million since the formation of the state in 1949 (Cai, 1988).

#### Regulatory and Pharmacopoeial Developments

The establishment in 1985 of the China State Administration of Traditional Chinese Medicine began the formal organization of TCM research and development nationally and internationally, sowing the seeds for the formal cooperative global links that would provide the backbone for the future of international Chinese medicinal plant research. China’s motivation to secure international links was also manifest in the publication of the PRC’s first dual Chinese and English language Pharmacopoeia, ChP, 4^th^ edition in 1997, which began its new 5-year publication cycle trend.

#### Medicinal Plant Research and Analytical Developments

The newly fostered R&D investment and cooperation during this period globally is represented by the leap in sophistication and complexity of the research published, with a shift from basic to more advanced biochemical investigations and more emphasis focused on disease and diagnosis strategies such as in cancer and infectious disease. The most widely cited articles of this time include advanced biomedical research on Forskolin, from the roots of *Plectranthus barbatus* Andrews as a diterpene activator in nucleotide metabolism. Even though basic biochemical equipment and colorimetric methods and spectrometric enzymatic assays were used, a more complex understanding of plant metabolites is apparent (Seamon et al., 1981).

This is also evident in the investigation of lectins as cell recognition molecules and their involvement in a wide range of molecular processes and potential pathologies, e.g., in metabolic regulation, viral, and bacterial infection processes (Sharon and Lis, 1989). In addition to plants playing a role as phytochelants in complexing heavy metals (Grill et al., 1985 and Grill et al., 1987), licorice was studied in greater depth using a conceptually new approach of assessing the mineral-corticoid activity of licorice and its role in sodium retention (Stewart et al., 1987) and the radical scavenging properties of its flavonoids (Hatano et al., 1988).

Awareness of plants having a role in cancer with both causative and curative effects emerged, with a highly cited review of potential causes of esophageal cancer in China. Particular concerns were linked to effects of fungal growth and associated nitrosamines due to poor storage conditions (Mingxin et al., 1980). This was a precursor to later studies on aflatoxins, which are now acknowledged as causing serious health problem linked to poor storage and processing. From a therapeutic perspective, the interest in antileukemia and anti-tumor agents, e.g., in *Taxus brevifolia* Nutt. stem bark, first investigated some decades before, continued and ultimately resulted in the introduction of a completely new therapeutic approach (Wani et al., 1971).

One of the landmark discoveries in medicinal plant history was reported to the west during this period. The antimalaria effect of artemisinin, derived from *Artemisia annua* L., for which the Chinese scientist Youyou Tu later received a Nobel Prize in Medicine (Klayman, 1985), described a conceptual shift in the approach to treating malaria, illustrating both a change in approach from using quinoline-based drugs, which parasites were showing increasing resistance to, and paving the way for the development of new classes of drugs e.g. with potential in antiviral and anticancer treatment (Su and Miller, 2015).

### 1990–2008

#### Political Context

This period in China was characterized largely by economic, political, and academic success delivering on the earlier aspirations of Deng Xiaoping through focused planning and the tight administrative grip of three successive presidents (Chairpersons) and state administration. An unusually high-performing economy producing more than a 10% sustained gross domestic profit (GDP) created a stable base for China to successfully join the world trade organization in 2001, marking its arrival on the world stage as a competent economic power and its transition to a market economy (Morrison, 2013). This, however, came with challenges to families and the environment.

On a local level as communes of the last decades had dissolved, a system of “household responsibility” was adapted as a kind of contract that guaranteed agricultural family holdings to provide a certain level of food (and herb) output (Ash, 1988). This ensured that levels of agricultural production were optimized for the land available. Because families were now allowed to sell grown products in an open market that mirrored the economic national trend, food and medicinal herbs began to take on more distinct financial attributes. This combined with mass migration of rural workers to rapidly developing industrialized cities away from countryside homes without sufficient locally produced food in urban surrounds created a situation of widespread supply and demand, leading to new value chains for food and medicinal plant products, along with potential motivation for the substitution or adulteration of these products.

#### Regulatory and Pharmacopoeial Developments

As industrialization occurred so too did environmental pollution, with increased volume and concentration of raw materials and waste presenting greater potential for pollution of medicinal plant material. The PRC at this stage had gone through a period of prolonged political stability. Economic policy became more flexible and governance developed an increasingly regulatory role compared with that of previous, more rigid enforcement. Regulation and safety testing of medical products saw further guidance through the production of four further volumes of the ChP in both Chinese and English culminating in the 8^th^ edition in 2005, listing 3,217 monographs, almost double that of the 1990 edition. This period saw China’s confidence increase and extend to regulatory and guidance aspects, with the ChP undergoing the greatest leap in analytical sophistication and rate of change to date. The 1990 edition was a significant step in the acceptance and introduction of modern instrumental analytical techniques for standard herbal substance testing. Since the 1985 edition, specific identification tests were introduced using mainly thin layer chromatography (TLC). Now chromatogram images of the crude and test samples were included and required for testing. Basic identification was expanded to require quantitation where high-performance liquid chromatography (HPLC) and GC were now included for the first time and TLC extended for content analysis. More instrumental techniques replaced older ones such as the introduction of spectrophotometric determination of the alkaloid content of berberine, which had been gravimetrically analyzed in previous editions. Quantification moved from measuring simpler marker components to more specific active compounds like anthroquinone from He Shou Wu, *Polygonum multiflorum* Thunb [now *Reynoutria multiflora* (Thunb.) Moldenke]. The 2000 edition introduced assays for residues of organic chlorine pesticides for Gan Cao, *Glycyrrhiza uralensis* Fisch. ex DC. and Huang Qi, *Astragalus membranaceus* Fisch. ex Bunge (Kwee, 2002). Another leap occurred in the 2005 edition with an expansion of the acceptance of HPLC-MS, LC-MS-MS, and DNA molecular markers and chemical fingerprinting, setting the stage for 21^st^ century pharmacopoeial trends and the ChP as a central global influence for the analysis of medicinal plants.

#### Medicinal Plant Research and Analytical Developments

The fruition of investment in external academic relations from the “opening up” phase and internal support for the now formed TCM structures of the previous decades state initiatives were borne out by the publication output in this period, with a six-fold increase in output compared with that of the previous equivalent 20-year period. Much of the output from this time demonstrated a refinement of thought around the effect of plant compounds on humans as a holistic system rather than the more singular metabolic pathway thinking of previous years. It also shows a tremendous emphasis on obtaining large datasets especially of the known metabolites and a wide exploration of acclaimed effects. Whole plant extracts and combinations of metabolites rather than single ones became a core theme, as became a medicinal plant’s effect on longer term health and preventative medicine. This ignited a resurgence of interest in the analysis of medicinal plants as a source of lead compounds for drug discovery.

The role of medical plants in coronary disease analysis becomes topical during this phase, e.g., long-term studies on elderly demonstrating the reduced risk of death from sustained flavonoids intake *via* inhibition of the oxidation of low-density lipoprotein (Hertog et al., 1993). More sophisticated quantitative analysis and differentiation appeared during this time such as HPLC of mulberry leaves containing four varieties of flavonoids (including rutin and quercetin), and their antioxidant properties (Zhishen et al., 1999). Flavonoid coronary disease risk prevention and cancer roles were advanced by the characterization and analysis studied in a wide range of fruits, seeds, oils, wines, and tea (Middleton et al., 2000). A greater awareness of the potency and efficacy of drugs and medicinal plants became evident as in the studies and analysis of the effect of fluorine on drug binding and potency (Purser et al., 2008). Cancer research also demonstrated further advances through combining previous findings on receptor binding with advancements in DNA extraction, amplification techniques, and cloning techniques. Resveratrol became a key area of interest for its chemoprotective effects (Jang et al., 1997).

Many of the most cited publications of these two decades were detailed reviews, which brought together the findings of previous research on individual plant research.

### 21^st^ Century

China’s growing influence was marked in 2011 with the Chinese State Administration of TCM (SATCM) forming an official relationship with the European Directive on the Quality of Medicines (EDQM) to share expertise and knowledge in addition to raising the standards of testing in China and Europe through cooperation. These include translation of historical TCM documents, information relating to preparation of products, process, and sourcing. Europe, seen as an aggregate, has an approximately 16% representation in the last decades’ research output, higher than the USA. The European Pharmacopoeia (Ph Eur) manages CHM’s by allowing importation of CHM’s to countries who have signed up to the European Pharmacopoeia convention. Currently there are 43 CHMs included in the Ph Eur, 8th edition, 34 from the Ph Eur TCM Working Party, 21 of which have been included as full monographs (Wang and Franz, 2015). New Ph Eur CHM monographs are being developed based, in part, on the ChP. This was facilitated by a working party on TCM (Ph Eur WP) and was officially introduced in 2005. It included 38 member states with a delegation from the EU (a representative from DG Health & Food Safety and the European Medicines Agency). Additional observers are composed of 27 countries/regions/organizations [which include 7 European countries, the Taiwan Food and Drug Administration (TFDA), and World Health Organization (WHO)] (EDQM, 2017). The WHO, through participation in the PhEur, additionally has led efforts to develop a harmonized international pharmacopoeia (WHO, 2018).

The monographs for medicinal plants in Ph Eur have developed from standard western drug monographs with an emphasis on chemical and physical testing, while those in the ChP have formed from revisions of older traditional texts.

As pharmacopoeial monographs expand and develop, so too does the range and complexity of analytical methods and analytical hardware needed to meet the regulatory demands and expectations of quality.

These emerging research trends and pharmacopoeial directives have paved the way for the development of a broad range of analytical techniques, mainly centering around the use of liquid chromatography (LC), GC, MS, and established UV/visible spectrophotometric techniques.

We present a selection of these analytical techniques and give examples of their applications in the analysis of medicinal plants and medicinal plant products.

### Analytical Hardware, Attested and Emerging Methods

#### High-Performance Liquid Chromatography

HPLC is one of the most developed and widely used analytical techniques. It is built on a historical knowledge base amassed from TLC and optical chemistry experience. HPLC chromatography elements rely on similar principles of TLC/HPTLC, where separation of components is dependent on selective affinities to stationary supports and liquid phases.

Detection employs a photomultiplier system able to detect individual wavelengths of light, a range (spectrum) and/or multiple simultaneous wavelengths in its different iterations, combined in an enclosed automated instrument system with sample injectors; this has significantly increased the precision and reproducibility of the chromatography when compared with older chromatographic methods. The widespread use of HPLC has made it more affordable for laboratories. High operator skill level is not required; it is robust and sensitive to low level detection and is particularly used for the quantification of components (active substances and adulterants).

HPLC applied to herbal products is well developed, and it has been successfully applied to the analysis of complex mixtures of similar compounds, both for the separation of individual compounds and for the differentiation of medicinal plant species. The high resolution of the technique has supported the development of the concept of a characteristic “fingerprint” developed for medicinal plants and herbal products to aid identification and authentication, e.g., Li et al. (2010) demonstrated differentiation of the same type of medicinal plant product from 40 different manufacturers, while simultaneously separating nine marker chemical compounds (berberine, aloe-emodin, rhein, emodin, chryso- phanol, baicalin, baicalein, wogonoside, and wogonin).

#### High-Performance Thin Layer Chromatography

HPTLC has become a common addition to the method section of new monographs, replacing the widely used TLC tests; it has shown to be a reliable and reproducible method of analysis that provides essential information regarding the compositional quality of an herbal substance.

Some advantages of this technique include low cost and a relatively simple test method. It does not require advanced sample preparation methods or high levels of expertise. Sample amounts are relatively small, and it is a more sensitive technique compared with HPLC, well suited to detecting contaminants. However, some disadvantages are that the reproducibility is dependent on a variety of external factors, and although more sensitive than HPLC, it is not able to sufficiently detect compounds at very low concentrations (PPB) where LC-MS (or HPTLC-MS) may be more suitable. HPTLC relies on the same principle as TLC and uses similar TLC plates and mobile phases, although relatively small amounts of solvents are required compared with standard TLC. The process of adding the sample to plates (spotting) has been made more reproducible and precise by spraying the sample onto the plate to form a band of compound rather than a spot. Retention factors for individual compounds are more reproducible due to controlled humidity during development. Derivatizing the analysis plates is completed mainly by machine and the visualization is captured by modern camera systems connected to powerful software. The software allows further manipulation of images to optimize visualization in a way that would be very difficult chemically. Another advantage is that the HPTLC system can be easily linked to a scanning densitometer; this not only allows for more precise quantitative work to be carried out but also the data can be exported for multivariate analysis. It is likely that more of the monographs with TLC requirements will be upgraded to HPTLC in the future.

#### Gas Chromatography

GC in respect to medicinal plant analysis is mainly used for the analysis of compounds with higher volatility, e.g., compounds found within essential oils, and more volatile adulterants, e.g., pesticides. While single GC column chromatography and its hyphenated derivatives have been use for many years, 1991 saw the introduction of 2D-GC or GC x GC, where the eluents of a standard separation are trapped and recirculated for another round of separation. This allows not only greater resolution and better separation but also the ability to purge undesired or interfering compounds so that more specific areas of the separation can be targeted (Liu and Philips, 1991). This led the way for multidimensional gas chromatography (MDGC) and the advances of the modules and valve systems that trap, control, and divert sample streams. These improvements extend to the thermal control and valve systems allowing greater thermal flow and split streaming (Bahaghighat et al., 2019). One key problem with GC is the introduction of sample into a gas stream. Historically squeezing, boiling, and later distillation of herbal materials were used for the collection and production of volatile compounds such as oils. However, the inherent instability of volatile components and losses as well as the poor recovery of these substances presented difficulties. This situation has somewhat been overcome by advances in extraction techniques such a solvent-free microwave extraction, e.g., for citrus peel oils [Citrus sinensis (L.) Osbeck]. No solvents or water are necessary for high recoveries with this method, and it allows for highly efficient, compatible sample introduction without the need for interfering solvents (Aboudaou et al., 2018). This sample extraction method commonly known as headspace analysis for GC has undergone many iterations (Gerhardt et al., 2018). It has now developed to the stage where it is increasingly used for bacterial and microorganism detection such as in *Commiphora* species (Rubegeta et al., 2018).

Microextraction techniques are essential for the introduction of small sample volumes into the GC gas stream. Needle-based extraction techniques have the advantage of automation, ease of interface to other instruments, and compatibility with miniaturization. Advances in solid phase dynamic extraction (SPDE), In-tube extraction (ITEX), and needle trap extraction (NTE) have refined the use of these techniques for natural and herbal compounds (Kędziora-Koch and Wasiak, 2018), e.g., SPDE and ITEX for pesticide residues in dried herbs (Rutkowska et al., 2018), herbal mint aromas compounds in commercial wine (Picard et al., 2018), and volatiles in Chinese herbal formula Baizhu Shaoyao San (Xu et al., 2018).

#### Supercritical Fluid Chromatography

Another liquid-based chromatographic technique based on pressurized low viscosity (supercritical) fluids, often carbon dioxide, is supercritical fluid chromatography (SFC). Since its introduction by Klesper in 1962, it has made large advances mainly due to improvements in its initially troublesome instrumentation (Desfontaine et al., 2015). Its main advantage over other techniques is in its usefulness for separating complex components characteristic of natural compounds. Selection of the correct conditions of SFC mobiles phases and modifiers can be finely tuned across a wide range of polarities from non-polar to polar allowing a broad selection of separations (Gao et al., 2010). Early analysis of natural products with SFC was when it was first hyphenated with gas chromatography (King, 1990). Recently, it has been more fully developed to analyze a range of natural compounds in herbal substances, notably, focusing on terpenes, phenolics, flavonoids, alkaloids, and saponins. This has been achieved with hyphenation to MS, diode array detectors, SFC-ELSD, in addition to the development of novel stationary phases such as cyanopropyl, pentaflouro phenyl (PFP), and imidazolyl. An example of this is with the separation of coumarins in *Angelica dahurica* (Hoffm.) Benth. & Hook.f. ex Franch. & Sav. roots and anthraquinones in rhubarb root (Pfeifer et al., 2016).

#### Near-Infrared Spectroscopy

Although commonly used within industry since the 1990’s, near-infrared (NIR) spectroscopy was not the method of choice for medicinal plant analysis mainly due to overlapping peaks making interpretation of data problematic, and consequently, it never became the instrumentation of choice within the quality control laboratory in the same way that HPLC and TLC developed. However, with the addition of new computational software, NIR is re-emerging as an affordable and useful analytical technique used in the analysis of medicinal plants and has been particularly favored by Chinese companies in routine quality control analysis due to its ability to both rapidly differentiate between species and provide quantitative information on metabolite content (Li et al., 2013; Zhang and Su, 2014).

As with HPTLC and NMR data, NIR also provides an opportunity for multivariate analysis and it appears capable of resolving very small variations in metabolite content. It is argued that more traditional TLC or HPLC techniques can be more subjective in the data interpretation stage and require a high degree of operator skill and that NIR is more suitable for high volume analysis in the routine quality control laboratory (Wang and Yu, 2015). However, this has partly been addressed by the introduction of the fully automated systems available for HPTLC analysis and the inclusion of scanning densitometry equipment that reduce the need for operator interpretation. The main advantages of NIR appear to be the preservation of sample integrity, little sample preparation needed, and no need for solvents, and it has shown to perform well comparable to HPLC for species differentiation and quantification of metabolites (Chan et al., 2007). Probably the main drawback in NIR compared with other methods, and especially, TLC, HPTC, LC-MS is in its sensitivity and some reports suggest that this technique may only be suitable for detecting compounds that exist at a concentration above 0.1% (Lau et al., 2009). Another consideration is that variation in NIR data is dependent both on the chemical and physical properties of the sample, with the physical properties, e.g., particle size, having greater effects on the variation than the chemical. Therefore, before multivariate analysis can take place some pre-treatment of the spectral data is necessary, e.g., to reduce baseline noise, light scattering, and consequently enhance any chemical variation in the sample set (Chen et al., 2008). Some advantages of NIR certainly are apparent, although it may not be appropriate for all situations and all types of samples. The technology has made a huge leap forward since its first introduction and now it needs to establish itself more widely as a useful tool in the quality analysis of medicinal plants.

#### Hyphenated Techniques

Combinations of techniques with modern developments in metabolomic analysis and computational pattern recognition programs open up a wider scope of applications to medicinal plant analysis. Tandem combinations of analytical instrumentation such as MS with HPLC has proved a productive route to expanding analytical medicinal plant applications. Not only in identification and fingerprinting but further chemical characterization of individual compounds e.g., Liu et al. (2011), characterized a spectrum of alkaloid components in the Chinese herb Ku Shen (*Sophora flavescens* Aiton). Further combinations and permutations of MS and NMR in combination with HPTLC have been demonstrated, such as the detection of acetylcholinesterase inhibitors in galbanum in a search for natural product drug candidates (Hamid-Reza et al., 2013), and mass spectroscopy (MS) HPTLC-MS shown for *Ilex vomitoria* Aiton with the use of a sampling probe following HPTLC combined with MS with Electrospray Ion Trap (Ford and Van Berkel., 2004) and *Hydrastis canadensis* L., with HPLTLC-MS atmospheric pressure chemical ionization (Van Berkel et al., 2007).

Analytical combinations including ESI-IT-TOF/MS-HPLC-DAD-ESI-MS have been demonstrated for the analysis of coumarin patterns in Angelica polymorpha Maxim. roots (Liu et al, 2011) and multihyphenated techniques such as SPE-LC-MS/MS-ABI quadrupole trap have been used for the analysis of six major flavones in *Scutellaria baicalensis* Georgi (Fong et al., 2014) and 38 saponins in the roots *of Helleborus niger* L. by LC-ESI-IT-MS (Duckstein et al., 2014).**

Merging the separation ability of HPTLC or HPLC with the analysis power of NMR and MS has significant benefits for analyzing complex samples in complex matrices such a blood, soil, and plants. However, each technique also possesses its inherent disadvantages. MS being complex, expensive, and time-consuming, requiring high analytical skill levels, it may not be suitable for a general quality assurance laboratory. Though powerful, extensive method development and post analysis data processing is required when applied to natural compounds with broad complex compositions in contrast to simpler synthesized pharmaceutical ingredients. Similarly, NMR is also expensive and sensitive to variations in sample preparation and composition. It is not fully applicable to all natural compound samples and signals generated from NMR analysis often overlap making data analysis for individual compounds problematic. However, the relative speed, rich information output, and insight into the overall composition of medicinal plants from both MS and NMR far outweigh the disadvantages. These techniques allow the detection of compounds into the parts per billion analytical range (MS) and allow a detailed fingerprint of metabolites across differing polarities (NMR) and so for research and for larger companies they are highly applicable analytical hardware.

## Metabolomics

Pharmacopoeial methods focus on authentication and quality of herbal materials; however, metabolomics allow us to go a step beyond authentication and look in more detail at a broad range of secondary metabolites. By coupling analytical data to multivariate software, this allows us to develop statistical models to firstly differentiate between species but also to get a better idea of a typical metabolite composition for a particular species. The advantage of this is that it can help to inform any laboratory test or clinical intervention. There has been great emphasis on making sure that any experiment or intervention uses plant material that is authenticated, with a herbarium specimen deposited. However, the requirements do not stipulate that a good representative of the species should be used. This is where metabolomics can provide essential information—by collecting a wide range of samples from different geographical locations, altitudes, growing conditions, it allows us to map their metabolite differences and highlight how diverse or how similar metabolite composition is. When an experiment is performed, we have the choice to use a specimen that may be typical, i.e., contains an average composition or we can look at compositions that are atypical, containing greater amounts of specific metabolites or even different metabolites. Moreover, if a particular experiment produces positive results and we want to reproduce the data, a metabolomic model allows us to choose species that have a similar composition.

This approach has important economic implications as a detailed understanding of metabolomic analysis allows us to inform industry as to how to grow plants that will be of the best composition and so help to support local livelihoods of farmers and primary processors in developing economies, e.g., Chachacoma (*Senecio nutans Sch. Bip.*) cultivation in the high altitude regions of Chile where metabolomics has helped to establish the best altitude for growing plants with the highest content of the anti-inflammatory acetophenone (Lopez et al., 2015).

This strategy also has applications in product development, where metabolomics can help to determine the quality of products based on their metabolite content, e.g., *Curcuma longa* L. (Turmeric products) (Booker et al., 2014), and also help to provide evidence that can lead to value addition of a product and greater confidence in its quality and safety.

## Nanoparticles

Nanoparticles 1–100 nm sized ions or organic/inorganic molecules have proven to be important in the development of new analytical testing (Tao et al., 2018), occupying the analytical regions of space between the ionic dimensions and small molecules.

Recent developments in nanoparticle research has led to an increased focus on chemo-bio sensing, as DNA has become the most used biological molecule to functionalize nanoparticles. Nanoparticles have provided many advantages to more consistent and specific testing including providing a more reproducible stable matrix for research and development, more controllable and reliable basis for designing and conjugating to functional molecules, and a wide rebate of flexibility for purification, selection, and modification of analytes. Nanoparticles have been used in creating a biological bar code for trace analysis of mycotoxins in Chinese herbs e.g. conjugated nanoparticles with DNA fragments to bind and target Chinese medicinal plants, e.g., Jue Ming Zi [Cassia seeds—*Senna obtusifolia* (L.) H.S.Irwin & Barneby], Yuan Zhi (*Polygala tenuifolia* Willd.), and Bai Zi Ren [*Platycladus orientalis* (L.) Franco] (Yu et al., 2018).

## The Future

The next steps in analytical advancement in combination with technological improvements will most likely occur in the realm of artificial intelligence. Neural networks have already shown promise in consumer electronics and online search engine optimization. Self-learning algorithms have been in development for decades, with great potential for the application of self-synthesizing, auto-creating, and auto-adapting algorithms, which can optimally recognize and synthesize analytical data into meaningful and useful patterns. This goes beyond what a single human mind could hope to achieve in lifetimes, now possible in seconds with current and more so with future technology. This extends not only the human potential of thinking and observation but also prediction and design. This could potentially play a role in self-design of analytical instrumentation and its modules, self-optimizing of methods in real-time, saving time that would perhaps take an analyst weeks or months of human work-hours to complete.

The greatest challenge with AI is its opacity and computational complexity. With self-learning systems already self-generating codes and pathways that would take decades for a single human to decode and understand, if ever possible. This presents a great challenge for use in reproducible, validated quality-driven, audit-trailed regulated orientated environments. This is where natural compounds such as herbal substances can play a significant role i.e. data from the same plants species with variable composition can help verify the input and outputs of complex analysis and recognition software. In AI-driven systems, natural substances are ideal candidates for testing the analytical attributes such as accuracy, precision, and robustness of whole AI-instrumentation systems.

## Conclusions

As pharmacopoeial requirements continue to develop and instrumental technology advances, it is clear that we will be able to delve further and further into the chemical composition of medicinal plants and develop more advanced techniques for the detection and quantification of adulterants and contaminants. However, it should be considered that although these technological advances give us this opportunity, more traditional organoleptic analysis also provides us with essential sensory information regarding medicinal plant quality.

We have shown the emergence and historical importance of complex analytical techniques used in medicinal plant analysis. However, any analytical approach, can only provide a partial perspective on complex multicomponent preparations. So future improvements in this area may not entirely rely on developing ever more complex analytical techniques, but in implementing best practice throughout all stages of the production and supply of herbal medicines.

## Author Contributions

AB wrote the sections on applications of metabolomics, NIR, parts of the introduction, and conclusions. MF wrote most of the instrumentation, trends in publications and history, part of the introduction and conclusions. MH contributed towards the methodological design of the study and assisted with the data analysis.

## Funding

MF scholarship is funded by Brion Research Group (Sun Ten Pharmaceutical Co) and Herbprime, UK.

## Conflict of Interest

MF scholarship is funded by Brion Research Group (Sun Ten Pharmaceutical Co) and Herbprime, UK.

The authors declare that the research was conducted in the absence of any commercial or financial relationships that could be construed as a potential conflict of interest.
